# THE EFFECT OF ENACTED AND PERCEIVED SOCIAL SUPPORT ON THE WELL-BEING OF KOREAN OLDER ADULTS BY GENDER

**DOI:** 10.1093/geroni/igad104.2200

**Published:** 2023-12-21

**Authors:** Meeryoung Kim

**Affiliations:** Daegu University, Seoul, Republic of Korea

## Abstract

Social support is important for the well-being of older adults. However, different kinds of social support have different effects on well-being. This study investigated enacted social support which is “the actual use of social support,” and perceived social support, in which people think they can get help when they need it. In short, enacted social support is action-oriented. Perceived social support is experience-oriented. For the effects, we looked at whether there were gender differences in the well-being of older adults. This study used the seventh additional wave (2018) and eighth wave (2019) of the Korean Retirement and Income Study. The subjects of this study were 60 and older. The sample size of this study is 4,338. Multiple regressions were used for data analysis. Demographic variables were controlled. This study used enacted social support and perceived social support as independent variables. Health and life satisfaction were used as well-being variables. Research shows that enacted emotional support is important for health. However, perceived emotional support is only significant for men, and perceived instrumental support is only important for women. All kinds of social support affected older women’s life satisfaction. For men, however, instrumental support did not affect life satisfaction. These findings imply that emotional support, whether enacted or perceived, is important for the health of older adults. Except enacted instrumental support, every support was significant for older adults regarding life satisfaction. Therefore, when providing social support, it is important to consider what type of support is effective for each gender.

